# The association between adverse childhood experiences and alterations in brain volume and cortical thickness in adults with alcohol use disorder

**DOI:** 10.1111/adb.13438

**Published:** 2024-09-19

**Authors:** Cagdas Türkmen, Haoye Tan, Sarah Gerhardt, Emilie Bougelet, Maria Bernardo, Noah Machunze, Yasmin Grauduszus, Maurizio Sicorello, Traute Demirakca, Falk Kiefer, Sabine Vollstädt‐Klein

**Affiliations:** ^1^ Department of Addictive Behaviour and Addiction Medicine, Central Institute of Mental Health, Medical Faculty Mannheim University of Heidelberg Mannheim Germany; ^2^ Departamento de Física, Faculdade de Ciências, Universidade de Lisboa Lisbon Portugal; ^3^ Department of Neuroimaging, Central Institute of Mental Health, Medical Faculty Mannheim University of Heidelberg Mannheim Germany; ^4^ Department of Psychosomatic Medicine and Psychotherapy, Central Institute of Mental Health, Medical Faculty Mannheim University of Heidelberg Mannheim Germany; ^5^ Mannheim Center for Translational Neuroscience, Medical Faculty Mannheim University of Heidelberg Mannheim Germany; ^6^ Feuerlein Centre on Translational Addiction Medicine University of Heidelberg Heidelberg Germany; ^7^ German Center for Mental Health (DZPG), partner site Mannheim‐Heidelberg‐Ulm Mannheim Germany

**Keywords:** alcohol use disorder, child maltreatment, brain volume, cortical thickness, structural imaging

## Abstract

**Background:**

Previous studies have established a connection between adverse childhood experiences (ACE) and alcohol use disorder (AUD), both of which are associated with alterations in grey matter volume (GMV) and cortical thickness (CT). The current study aimed to assess the neurobiological impact of ACE specifically in the context of AUD, as well as the role of maltreatment type (i.e., abuse or neglect) and timing.

**Methods:**

Structural MRI data were collected from 35 adults with AUD (mean age: 40; 31% female) and 28 healthy controls (mean age: 36; 61% female). ACE were assessed retrospectively using the Childhood Trauma Questionnaire, and the Maltreatment and Abuse Chronology interview. Global and regional GMV and CT were estimated using voxel‐ and surface‐based morphometry.

**Results:**

Relative to the healthy controls, the AUD group had significantly reduced CT in the left inferior frontal gyrus, left circular sulcus of the insula and subcentral gyrus and sulci (cluster C1), and in the central sulcus and precentral gyrus (cluster C2). Within the AUD group, a reduction of CT in cluster C1 was significantly associated with higher severity of ACE and AUD. Type and timing analyses revealed a significant association between higher levels of abuse at ages 13 to 15 and reduced CT in cluster C1 within the AUD group.

**Conclusions:**

In adults with AUD, abuse experienced during early adolescence is associated with reduced CT in regions involved in inhibitory control, indicating the potential relevance of cognitive pathways in the association between ACE and AUD. Longitudinal studies are needed to confirm and expand upon current findings.

## Graphical abstract

Adults with alcohol use disorder (AUD) had significantly reduced cortical thickness in brain regions linked to inhibitory control, notably in the left inferior frontal gyrus, compared to healthy controls. Within the AUD group, this reduction was associated with higher levels of adverse childhood experiences (ACE), specifically abuse experienced during early adolescence (ages 13‐15). These findings highlight the need for longitudinal research to examine the role of cognitive pathways in the association between ACE and AUD, and the potential relevance of early adolescence as a sensitive developmental period.

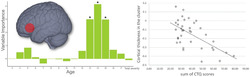

## 1 INTRODUCTION

Adverse childhood experiences (ACE), including emotional, sexual and physical abuse, as well as emotional and physical neglect and household dysfunction, remain a prevalent public health problem with a significant impact on premature mortality and socioeconomic burden.^1^, ^2^ ACE are common in the general population and have short‐ and long‐term lasting consequences on physical and mental health.^3^, ^4^ Notably, ACE increase the risk of developing substance use disorders (SUD) which rank among the psychiatric disorders with the highest mortality rates.^5^, ^6^, ^7^

Alcohol use disorder (AUD) has recently been reported to be the most prevalent SUD among German adolescents.^8^ The relationship between ACE and AUD was first studied by Felitti et al^9^ who found that adolescents who had experienced four or more ACE were at a 4‐ to 12‐fold increased risk of developing alcohol or drug abuse problems. In addition, a review of 12 retrospective studies found a higher prevalence of ACE in adults with SUD, compared to the general population.^10^ More recently, Broekhof et al^11^ have presented longitudinal evidence that adults with any history of ACE are approximately four times more likely to develop an SUD, confirming previous retrospective findings. This study also found sex‐specific risk profiles, showing that women were 5.9 times more likely to develop an AUD, whereas men were 5 times more likely to develop an illicit drug use disorder.^11^ Another study highlighted that particularly emotional abuse could explain an earlier onset of AUD among women.^12^ A history of childhood maltreatment is clinically significant in relation to AUD, as it can have an indirect impact on alcohol problems via emotion regulation difficulties.^13^ Moreover, research shows that women with AUD and a history of abuse exhibit lower abstinence rates than those without a history of abuse.^14^ Together, these findings highlight the need for clinical attention to AUD subgroups with exposure to ACE.

Although a risk association between ACE and AUD has been well‐established, potential mechanisms underlying this association remain to be elucidated. Given the susceptibility of the developing brain to neurobiological changes as a result of potentially traumatic experiences,^15^, ^16^ it is crucial to elucidate alterations in neural structures which may predispose affected individuals to increased addiction vulnerability. Previous studies have focused on the hippocampus and amygdala as particularly vulnerable regions to the deleterious effects of both early life stress and heavy alcohol use.^17^, ^18^, ^19^ Both the hippocampus and amygdala hold clinical significance in the context of AUD, given their role in memory formation^20^ and in emotion regulation,^21^ respectively.

Both ACE and AUD have been associated with a reduced volume in hippocampal subfields.^22^, ^23^ Regarding the amygdala, there appears to be a differential impact of ACE which is dependent upon type and timing.^24^ While neglect during childhood is associated with an increased amygdala volume,^25^, ^26^ abuse in later stages is associated with a decrease in amygdala volume.^27^, ^28^ In addition, previous studies assessing amygdala volume have been conducted in samples with varying types and degrees of psychopathology, which may account for differences in observed volumes.^24^ In the context of SUD, Van Dam et al^28^ have demonstrated that childhood maltreatment is associated with lower brain volume in the amygdala, hippocampus and other limbic structures, which were found to predict the severity of substance use relapse among patients with cocaine‐, alcohol‐ and cannabis use disorders.

The impact of ACE specifically in the context of AUD has been investigated in a study involving South African adolescents with AUD and healthy controls.^29^ The findings indicated that increased ACE severity was associated with reduced brain volumes in the right precentral gyrus and bilateral hippocampus in the AUD group, suggesting that the potential impact of ACE should be taken into account when studying neurobiological alterations in individuals with AUD.^29^ More recently, Soravia et al (2022)^18^ have demonstrated that childhood maltreatment is associated with decreased structural connectivity of the amygdala in patients with AUD. To build upon the current body of evidence, the aim of this cross‐sectional research was to investigate the neurobiological impact of retrospectively reported ACE in a sample of adults with AUD while exploring the role of ACE type (abuse or neglect) and timing using a machine learning approach. While prospective research better explains changes over time, the current research could provide valuable insights into relevant neural structures and sensitive developmental periods that may play a role in the development and maintenance of AUD. Such insights could guide future prospective studies. The following hypotheses and explorative research question were assessed:

**Hypotheses:**

1. Relative to healthy controls, adults with AUD have reduced voxel‐wise grey matter volume (GMV) and cortical thickness (CT), and at a region of interest (ROI) level, namely within the amygdala and hippocampus.
2. Within the AUD group, higher ACE severity is associated with a decrease in GMV and CT in relevant clusters from hypothesis 1 and in the ROIs (amygdala and hippocampus).
3. There is an interaction between ACE and AUD such that those in the AUD group with higher AUD severity show a stronger association between ACE severity and reduction in GMV and CT in relevant clusters from hypothesis 1 and in the ROIs (amygdala and hippocampus), compared to those with lower AUD severity.

Explorative research question:

4. Are specific types and timings of ACE associated with a decrease in GMV and CT in relevant clusters from hypothesis 1 and in the ROIs (amygdala and hippocampus) within the AUD group?

## 2 METHODS AND MATERIALS

### 2.1 Participants and procedures

Structural magnetic resonance imaging (MRI) data were acquired at the Central Institute of Mental Health (CIMH), Mannheim, Germany, between January 2019 and March 2021. The study was prospectively registered on ClinicalTrials.gov (Identifier: NCT03758053). The primary research questions and analysis intentions were not pre‐registered. The results should thus be considered exploratory. The ethics committee of the Medical Faculty Mannheim of the University of Heidelberg evaluated and approved the conduct of this study (ethics approval number: 2018‐560N‐MA). All study procedures were conducted in accordance with the Declaration of Helsinki. Written informed consents were obtained from all participants prior to data collection.

The sample comprised a healthy control (HC) group and an AUD group, including individuals with a diagnosis of AUD or heavy alcohol use, aged 18 to 65. Participants were recruited from public announcements mainly within the vicinity of Mannheim, Germany. In‐ and outpatients with AUD were recruited from the addiction ward and day clinic of the CIMH. Potential participants were screened by telephone to assess study eligibility, after which they were invited to complete two measurements, one baseline measurement and one MRI measurement.

During the baseline measurement, participants provided written informed consent, underwent a drug and pregnancy screening, and had their breath alcohol measured using a breathalyser. Sociodemographic as well as behavioural data (see Section 2.2) were collected. A diagnostic assessment was performed using the Structured Clinical Interview (4th version) of the Diagnostic and Statistical Manual of Mental Disorders (DSM‐IV) to assess psychiatric comorbidities, while the severity of AUD was assessed using the diagnostic criteria from the DSM‐V. Individuals were categorised into the AUD group if they either met the criteria for at least a mild AUD diagnosis or reported heavy alcohol use (alcohol consumption per day: ≥ 40 g (female), 60 g (male) on min. 5 days/week). A maximum of 28 consecutive days of abstinence was allowed within the AUD group. Exclusion criteria are specified in Table S1.

### 2.2 Measures

The severity of alcohol dependence was measured using the Alcohol Dependence Scale (ADS),^30^ a well‐established tool with supported validity and reliability.^31^ ADS scores range from 0 to 47, with scores of ≤ 13 indicating a low level of dependence, 14 to 21 an intermediate level, 22 to 30 a substantial level and 31 to 47 a severe level of dependence.

To retrospectively assess exposure to ACE, two measures were utilised, namely the Childhood Trauma Questionnaire (CTQ), a valid and reliable 28‐item self‐report questionnaire,^32^ as well as an adapted brief German version of the Maltreatment and Abuse Chronology of Exposure (MACE; German: KERF‐40‐I),^33^, ^34^ which was administered in an interview setting. The CTQ assesses five subscales of ACE, namely emotional, physical and sexual abuse, as well as emotional and physical neglect. Participants provided responses to each item on a five‐point Likert scale, ranging from “not at all” to “very often”, leading to sum scores between 5 (indicating no trauma) and 25 (indicating severe trauma) for each subscale. The KERF‐40‐I is a structured interview, which additionally considers the timing of ACE (i.e., age at occurrence of ACE between the ages of 3 and 17). A recent study has provided evidence supporting the validity and reliability of this adapted version of the instrument.^35^ Ten different ACE types were assessed, including emotional neglect and physical neglect, which make up the neglect score, while the abuse score comprises parental physical abuse, sibling physical abuse, parental emotional abuse, sibling emotional abuse, sexual abuse, peer abuse, witnessing interparental violence and witnessing violence to siblings. The scores for the single life years are the sums across different ACE events at a given age. Total ACE severity was calculated as the average score across these life years. ACE was quantified by a) an averaged KERF‐40‐I severity score indicating ACE across childhood and adolescence (i.e. global ACE severity), and b) by a KERF‐40‐I average for each year of life, respectively (i.e. time‐specific ACE severity). Both scores range from 0 to 100. Abuse is represented by collapsing all abuse subscales, while neglect is represented by collapsing all neglect subscales. The scores have been averaged across childhood and adolescence, i.e. global abuse severity, and global neglect severity, as well as for each year of life respectively, i.e. time‐specific abuse severity, and time‐specific neglect severity. The neglect and abuse scores each range from 0 to 20.

### 2.3 MRI acquisition and preprocessing

Structural MRI data were collected with a 3 Tesla whole‐body tomograph (Siemens PrismaFit, Erlangen, Germany). Images were obtained using a transaxial T1‐weighed image acquisition (voxel size 1x1x1 mm^3, FoV 232 x 256 mm^2, TR = 2000 ms, TE = 3.03 ms, TI 900 ms, flip angle = 9°). Voxel‐wise and regional GMV and CT were estimated using voxel and surface‐based morphometry (VBM and SBM) in the SPM12 software (The Wellcome Centre for Human Neuroimaging, at University College, London, UK) and its extension CAT12^36^ within the MATLAB environment (R2021, MathWorks Inc., Natick, Massachusetts). CT was estimated based on the projection‐based thickness (PBT) method.^37^ Preprocessing was performed using CAT12. All brain images underwent quality checks. Specifically, the ratio between the weighted overall image quality (IQR) and the mean correlation was examined using a boxplot. IQR combines measurements of noise and spatial resolution before preprocessing, while mean correlation assesses data homogeneity and image quality after preprocessing. This analysis was conducted separately for the AUD and HC groups and for volume and surface data, ensuring that anatomical differences from alcohol misuse did not skew mean correlation values. Low mean correlation by itself did not lead to exclusion, provided that image quality was sufficient. For surface data, the Euler number (indicating topology defects) and defect size, were additionally assessed. Based on findings from existing research,^17^, ^18^, ^19^ we defined bilateral amygdala and hippocampus as regions of interest (ROIs) in addition to whole‐brain voxel‐wise analyses.

### 2.4 Statistical analyses

Sixty‐nine individuals participated in the study. The analytical sample comprised *N* = 63 participants; *N* = 35 in the AUD group and *N* = 28 in the HC group. *N* = 6 participants were excluded from the analyses due to incidental neurological findings (*N* = 1), image quality concerns (*N* = 1), dropout before MRI assessment (*N* = 1), exclusion criteria (*N* = 1), MRI termination due to claustrophobia (*N* = 1) and MRI termination due to technical problems (*N* = 1). An overview of the study flow is provided in Figure S4. Psychometric data were analysed using SPSS (Statistics for Windows, Version 27.0., IBM Corp., Armonk, NY). Descriptive analyses as well as chi‐squared tests and *t*‐tests were applied to describe the sample and perform statistical analyses regarding group differences. CTQ scores were missing for one participant in the HC group.

Regarding the primary analyses, a two‐sample *t*‐test was used to assess voxel‐wise and regional differences in GMV and CT between the HC and AUD groups, controlling for age, gender and transcranial volume (TIV, only for GMV) (voxel‐wise‐*p* < .001 with cluster corrections by SPM random field theory, corresponding to pFWE < .05). The association between ACE and GMV/CT in the ROIs and relevant clusters from the voxel‐wise analysis was assessed using partial correlation, correcting for age, gender and TIV. In line with recommendations for MRI studies, only GMV (and not CT) was corrected for TIV.^38^ The Destrieux 2009 Atlas^39^ was used for the labelling of brain regions.

To identify sensitive life years for the effects of different ACE types (abuse or neglect) on neurobiological changes, we employed the same machine learning approach as Herzog et al,^40^ namely random forest regression with conditional inference trees. As our study incorporated 15 strongly correlated predictors (ages 3 to 17) into the same model, conditional random forest regression is particularly suited, given that it takes into account multicollinearity among predictor variables,^41^ and it has been frequently used in previous studies on sensitive periods for early adversity.^40^, ^42^, ^43^ For the analyses, we used the “cforest” function in the R package party^41^, ^44^ within the R software environment (Version 4.2.3, R Development Core Team 2022, Vienna, Austria). We ran the conditional random forest regressions for each ROI and extracted means from relevant clusters identified from the voxel‐wise analysis using randomly generated seeds to ensure the reproducibility of the results. Each ROI was corrected for age and TIV and then z‐transformed. Each random forest model consisted of 1,000 trees with four randomly selected variables at each split. To evaluate the predictive accuracy (performance) of each model, we present the explained variance and variable importance based on the out‐of‐bag samples. Each model was run separately for abuse and neglect (ACE type analysis). Models with a positive predictive accuracy were further used for permutation tests to identify predictors with statistically significant variable importance using 1,000 permutations. Given the exploratory nature, the *p*‐values in the permutation test were not corrected for multiple testing. For predictors with statistically significant variable importance, we performed follow‐up correlation analyses. The significance levels of all analyses were *p* < .05.

## 3 RESULTS

### 3.1 Descriptives

An overview of descriptive statistics can be found in Table 1. The AUD group, relative to the healthy control group, had a greater severity of alcohol dependence (ADS) and higher alcohol consumption in the past three months (FORM‐90). We also noted significant group differences in depression scores (BDI), gender ratio, years of education, smoking status and ACE severity (KERF‐40 and CTQ). Further details about the KERF‐40 subscales and an illustration of the average severity for abuse, neglect and overall KERF‐40 scores from the ages 3 to 17 are provided in Table S2 and Figure S1, respectively. An illustration of the distribution for CTQ subscore severities in both groups can be found in Figures S2 and S3.

**TABLE 1:** Sample description of AUD and healthy control (HC) groups.

	HC Mean (SD)	AUD Mean (SD)	Statistics
*N*	28	35	
Gender (male:female)	11:17	24:11	**χ** ^**2**^ **(1) = 5.4, *p* = .020**
Age (years)	36.3 (12.6)	46.5 (12.7)	T(61) = 1.32, *p* = .194
Marital status (married:divorced:single)	7:3:18	5:8:22	χ^2^(2) = 2.26, *p* = .324
Living status (alone:together with others)	7:21	14:21	χ^2^(1) = 1.57, *p* = .209
Years of education	15.8 (2.3)	14.3 (3.0)	**T(61) = −2.13, *p* = .037**
Smoker (yes:abstinent:no)	3:0:25	10:3:22	**χ** ^**2**^ **(2) = 6.26, *p* = .044**
KERF‐40 sum	5.1 (7.2)	19.3 (15.5)	**T(50.2) = −4.84, *p* < .001**
Abuse	0.5 (0.8)	1.8 (1.5)	**T(43.8) = −3.86, *p* < .001**
Neglect	0.5 (0.9)	2.2 (2.5)	**T(52.8) = −4.73, *p* < .001**
CTQ sum	32.1 (8.8)	39.4 (14.6)	**T(57) = 2.44, *p* = .018**
CTQ Emotional abuse	8.8 (4.9)	6.5 (2.8)	**T(55.5) = 2.36, *p* = .022**
CTQ Physical abuse	5.4 (1.1)	6.4 (2.5)	T(48) = 1.98, *p* = .053
CTQ Sexual abuse	5.1 (0.5)	5.5 (1.9)	T(38.7) = 1.15, *p* = .257
CTQ Emotional neglect	9.0 (5.0)	11.4 (5.5)	T(60) = 1.79, *p* = .079
CTQ Physical neglect	6.1 (2.0)	7.3 (3.3)	T(57.2) = 1.75, *p* = .086
ADS	2.4 (2.3)	11.9 (6.7)	**T(43.8) = 7.78, *p* < .001**
BDI	1.7 (3.2)	10.7 (8.8)	**T(43.5) = 5.52, *p* < .001**
FORM‐90 alcohol per day (g)	25.2 (19.3)	136.5 (125.8)	**T(35.9) = 5.16, *p* < .001**

*Note*: Significant group differences are highlighted in bold.

Abbreviations: ADS, Alcohol Dependence Scale; BDI, Beck's Depression Inventory; CTQ, Childhood Trauma Questionnaire; FORM‐90, amount of alcohol consumption per day over the last 90 days; g, grams; KERF‐40, Brief German version of the Maltreatment and Abuse Chronology of Exposure (MACE) interview; *N*, sample size; SD, Standard Deviation.

### 3.2 Hypothesis 1

**FIGURE 1:**
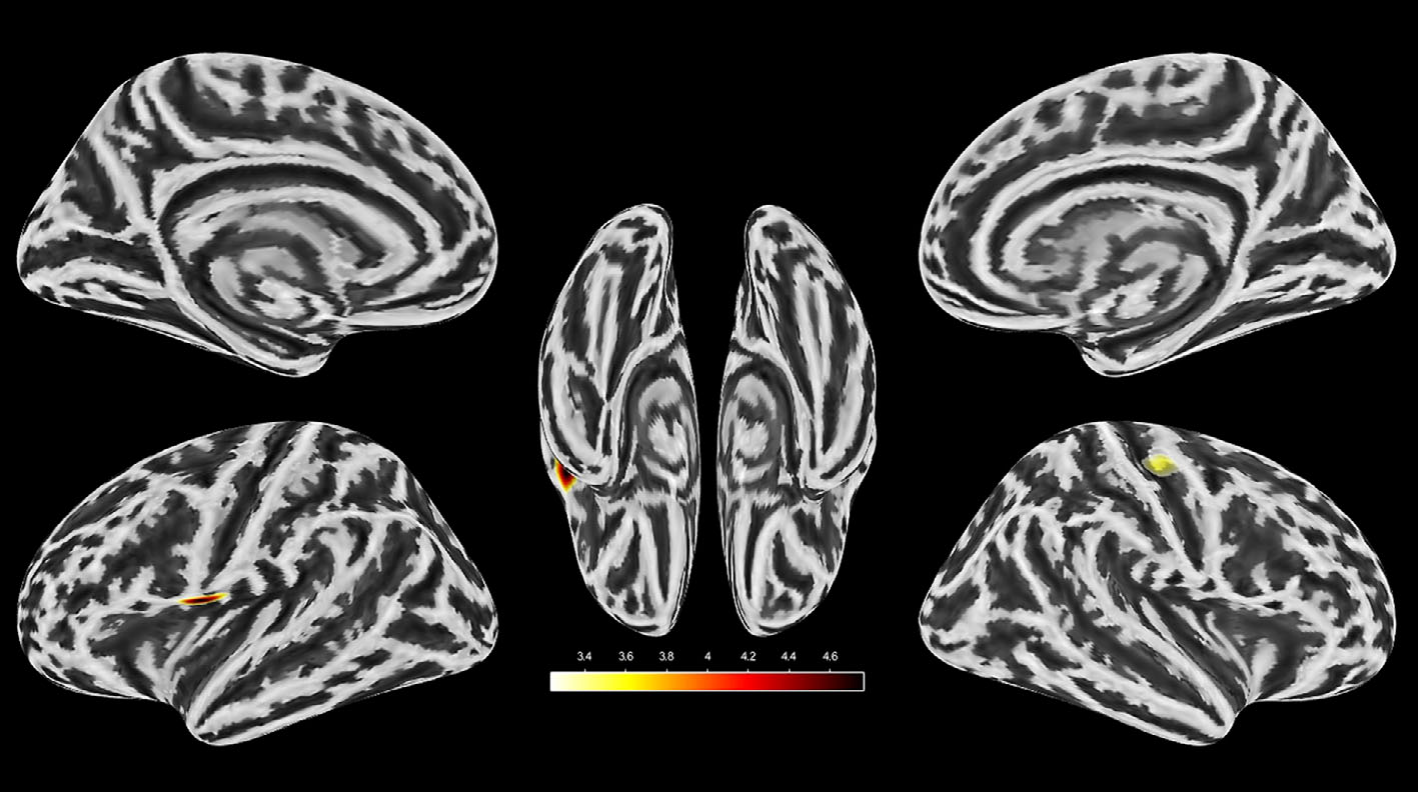
Significant clusters in the AUD group, relative to the HC group. A two‐sample t‐test was used with cluster corrections (voxel‐wise *p* < .001, cluster pFWE < .05). Reduced CT in the left inferior frontal gyrus, left circular sulcus of insula, and subcentral gyrus and sulci (cluster C1; size: 163 voxels, Peak T: 4.8, MNI: −42, 10, 5, *p* < .05) and in the right central sulcus and precentral gyrus (cluster C2; size 136 voxels, Peak T: 3.5, MNI: 42, −23, 40, *p* < .05).

The two‐sample *t*‐test indicated significantly reduced CT in the AUD group, relative to the HC group, in two clusters (see Figure 1); one cluster (C1) comprising the left inferior frontal gyrus (IFG), left circular sulcus of the insula and subcentral gyrus and sulci (cluster size 163 voxels, Peak T: 4.8, MNI: −42, 10, 5, *p* < .05) and a second cluster (C2) comprising the central sulcus and precentral gyrus (cluster size 136 voxels, Peak T: 3.5, MNI: 42, −23, 40, *p* < .05). No differences in GMV or in the ROIs were found.

### 3.3 Hypotheses 2 and 3

**FIGURE 2:**
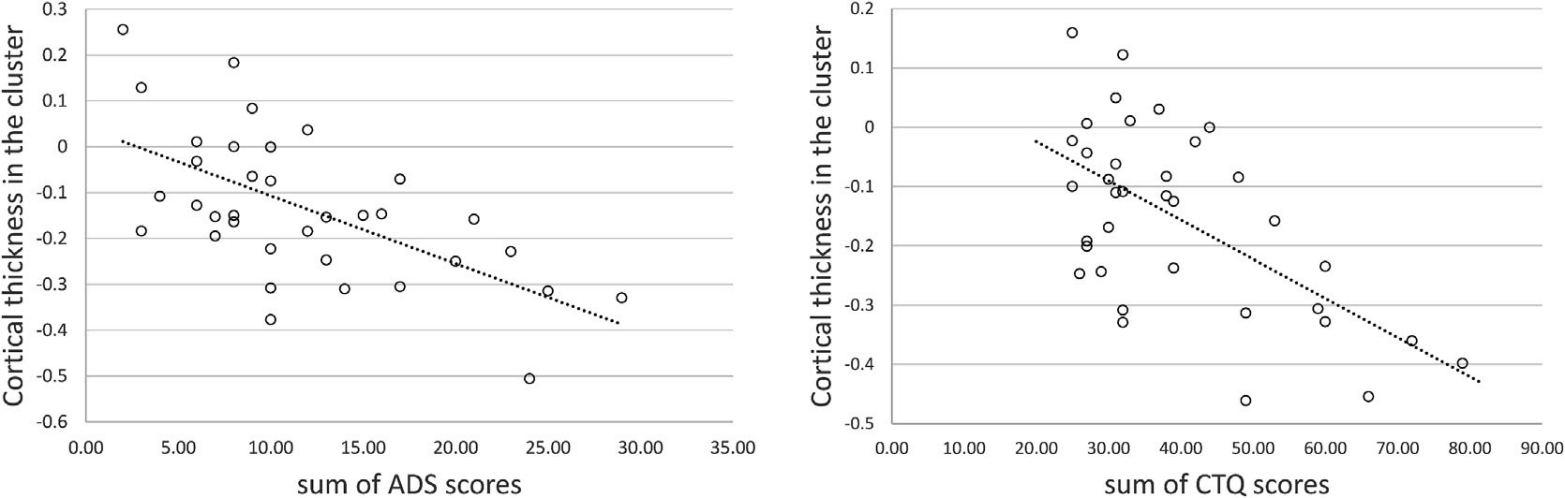
Partial correlation between CT in cluster C1 (see Figure 1) and ADS scores (left) and CTQ scores (right), controlling for age and gender.

There was a significant positive correlation between the CTQ sum score and KERF sum score in all participants (*r* = 0.765, *p* < .001). Total ACE severity was significantly higher in the AUD group compared to the HC group, as indicated by the CTQ sum score (*t*(57) = 2.44, *p* < .05) and the KERF‐40 sum score (*t*(50.2) = −4.84, *p* < .001). In the AUD group, there was a significant negative correlation between CT in cluster C1 and CTQ sum scores (*r* = −0.319, *p* < .05) as well as ADS scores (*r* = −0.346, *p* < .05; see Figure 2). There was no interaction between CTQ sum scores and ADS scores on CT in the clusters or in the predefined ROIs. No significant associations between CTQ sum scores and the ROIs or the other cluster (C2) were found.

### 3.4 Explorative research question (type and timing)

**FIGURE 3:**
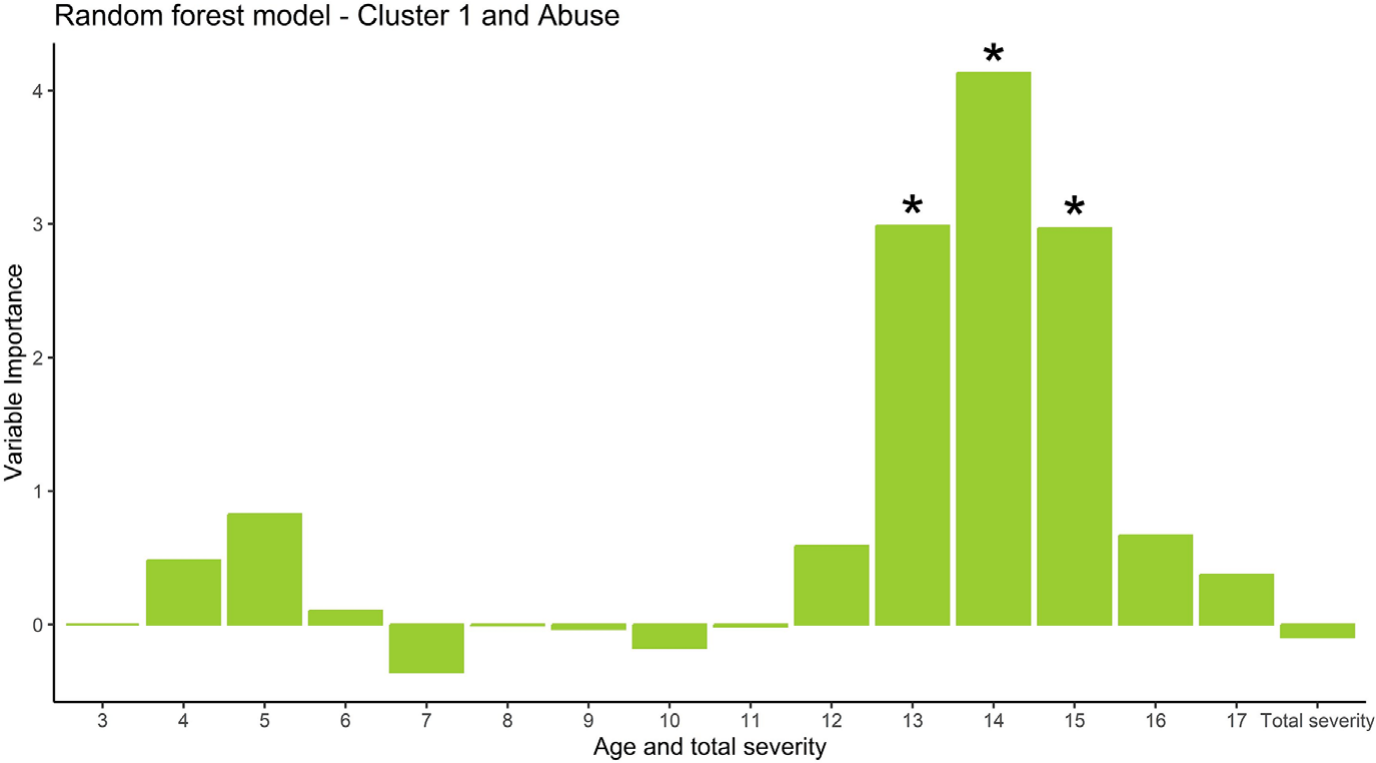
Random forest regression indicating the importance of time‐specific abuse severity from 3 to 17 years of age on cluster C1. *N* trees = 1,000; *N* randomly selected variables = 4. Permutation test: *p < .05; *N* permutations = 1,000.

The random forest regression revealed a low albeit positive predictive accuracy for a model with age‐specific and overall abuse severities (KERF‐40) as predictors and CT in the C1 cluster as an outcome (*R* ^*2*^ = 0.01). The permutation test of this model indicated statistically significant variable importance for abuse at the ages 13, 14 and 15 in the AUD group (*p <* .05, see Figure 3). Follow‐up correlation analyses revealed a significant association between higher abuse in these life years and reduced CT in the C1 cluster (age 13, *r* = −0.315, *p* < .05; age 14, *r* = −0.335, *p* < .05; age 15, *r* = −0.332, *p* < .05; FDR‐corrected). All other models had a negative predictive accuracy and thus were not used for further analyses.

## 4 DISCUSSION

The aim of this study was to assess the neurobiological impact of retrospectively reported ACE in adults with AUD and to explore the role of the ACE type and timing using a machine learning approach. The current results partially confirm our hypotheses, indicating that in the AUD group, CT was reduced and associated with higher ACE severity, whereas GMV analyses did not yield significant results. We found significantly reduced CT in a cluster comprising the left IFG, left circular sulcus of the insula and subcentral gyrus and sulci (C1), which was associated with a higher ACE severity. The random forest regression further revealed that greater abuse severity during early adolescence (ages 13 to 15) was significantly associated with reduced CT in this cluster. In addition, we found a second significant cluster with reduced CT in the AUD group, encompassing the central sulcus and precentral gyrus (C2). Unlike C1, reduced CT in this cluster was not associated with higher ACE severity. We found no interaction between ACE and AUD with respect to changes in the significant clusters or in the predefined ROIs.

Among the observed changes in CT, the finding that the AUD group exhibited reduced CT in the left IFG may be of particular importance with respect to the development and maintenance of AUD. This finding builds upon a previous study indicating that patients with alcohol dependence, relative to healthy controls, have reduced CT in the right IFG.^45^ While neuroimaging studies have indicated right‐hemispheric IFG dominance in inhibitory control,^46^, ^47^ there is evidence suggesting that the left IFG is also critical for response inhibition.^48^ In addition, the current results indicate that greater severity of alcohol dependence is associated with reduced CT in the IFG, consistent with a previous study that found a higher alcohol dependence severity to be strongly related to CT reductions in the inferior frontal region.^49^ This is further supported by findings from the Human Connectome Project showing that a greater drinking quantity and higher frequency of heavy drinking were associated with reduced CT in the IFG in young adult drinkers.^50^

Consistent with the findings related to AUD, recent meta‐analyses indicated that ACE are associated with decreased GMV and CT in the left IFG,^51^, ^52^ indicating the possible relevance of cognitive functioning in the association between ACE and AUD. This idea is supported by Edalati and Krank's model of cognitive pathways proposing that impairments in cognitive functions (e.g., response inhibition, working memory) mediate the relationship between ACE and SUD.^53^ As our type and timing analyses revealed that abuse during early adolescence is associated with reduced CT in the left IFG, it is possible that early adolescence represents a sensitive developmental period for abuse‐related cortical thinning, which may increase the risk of developing AUD. This possibility is supported by a review proposing that cortical development during adolescence is a crucial vulnerable period for addiction (for an overview, see review by Crews and Hodge^54^). In addition, the current findings converge with those of a recent longitudinal study showing that family‐specific negative life events were associated with increased alcohol use during middle school years.^55^ This supports the view that early adolescence may be a sensitive period for early life adversity‐related AUD development, as starting to drink before the age of 15 is associated with higher odds of developing AUD.^56^ Importantly, cognitive functioning could play an important role in this increased vulnerability. Indeed, a study by Gold et al^57^ reported deleterious effects of childhood abuse on CT in the parahippocampal gyrus in a sample of adolescents which, in turn, was associated with higher levels of externalizing psychopathology. This suggests that reduced CT in regions involved in cognitive functioning may serve as a risk factor for AUD in later life, although the underlying mechanisms remain to be elucidated. One line of explanation may lie in cognitive development being delayed following exposure to abuse^58^ and triggering a series of neurobiological alterations associated with cognitive deficits in adulthood.^59^

These findings hold clinical significance, as they underscore the potential relevance of cognitive deficits in the treatment of patients with AUD and a history of ACE. Previous research has indicated that cognitive deficits can arise during childhood following ACE and persist into adulthood, which may increase the risk of developing psychiatric disorders.^60^ These deficits align with cognitive impairments observed in individuals with AUD.^61^ Among the cognitive domains, executive functioning has emerged as a particularly relevant domain, as highlighted by a study revealing that deficits in response inhibition predict relapse in patients with alcohol dependence.^62^ In the context of ACE, Jankowski et al^63^ have provided initial support that maltreatment in early childhood is associated with changes in neural activation patterns underlying response inhibition during early adolescence. Moreover, their findings suggest that engaging in a preventive intervention could potentially mitigate the effects of maltreatment on the neural circuitry related to successful response inhibition. Thus, prevention and intervention programs should start early, and aim to incorporate response inhibition as a treatment target. Likewise, future longitudinal studies should incorporate cognitive functioning as an outcome, and assess changes at a neural level over time using neuroimaging‐based cognitive paradigms. Importantly, these studies should utilise treatments such as cognitive training and remediation interventions,^64^ and assess whether these interventions are associated with improved response inhibition or accompanied by structural changes in relevant brain regions.

In the present study, we did not replicate findings from a previous study by Van Dam et al^28^ indicating an association between higher ACE severity and reduced GMV in the amygdala and hippocampus in patients with SUD. Previous findings regarding the effects of ACE on GMV volumes in the amygdala and hippocampus have been mixed.^19^, ^52^ The AUD sample in the present research included many cases of minimally traumatised individuals, which is likely to have resulted from employing strict exclusion criteria with respect to psychiatric comorbidities. This might have led to range restriction with regard to ACE severity and thus diminished correlations. Indeed, in the study by Van Dam et al,^28^ SUD patients with childhood maltreatment had, on average, a considerably higher CTQ score compared to our AUD sample. In addition, several other limitations should be noted. First, this study had a modest sample size, which may have led to underpowered analyses to detect smaller effects of retrospectively reported ACE on GMV. It is unclear whether CT, relative to GMV, is a more sensitive outcome measure for assessing the influence of retrospectively reported ACE in AUD samples. Future prospective, longitudinal studies with larger sample sizes are needed to confirm and expand upon current findings. Second, while retrospective designs combined with machine learning techniques represent a fruitful approach to detect sensitive periods for ACE‐related addiction vulnerability, it should be noted that a recent meta‐analysis has reported low agreement between prospective and retrospective measures of ACE, raising concerns about whether these two measurement approaches capture different groups of individuals.^65^ Although retrospective designs introduce potential biases embedded within this methodology, studies employing prospective designs are likely to primarily include more severe cases of traumatisation. Third, due to the modest sample size, we did not examine potential sex differences regarding changes in GMV and CT. Given that different types of ACE have varying predictive values for developing SUD among men and women,^11^ future research should further elucidate potential sex‐ and ACE‐type‐dependent volumetric and cortical changes and their roles in the development and maintenance of AUD. In addition, while there were significant differences between the AUD and HC groups regarding years of education and smoking status, these were not incorporated as covariates of no interest. Specifically, due to the association of lower educational attainment and smoking with AUD and heavy drinking, this may remove AUD‐related variance and influence the effect of interest.^66^, ^67^, ^68^, ^69^ In our sample, it was not possible to disentangle the differential effects of education and smoking on the results. Finally, given the cross‐sectional nature of this study, there is no certainty regarding the directionality or causality of the findings.

## 5 CONCLUSIONS

In adults with alcohol use disorder, retrospectively reported abuse during early adolescence is associated with reduced cortical thickness in brain regions involved in inhibitory control, providing support for the potential relevance of cognitive pathways in the association between adverse childhood experiences and the development and maintenance of alcohol use disorder. Prevention and intervention programs should incorporate inhibitory control as a treatment target in individuals with alcohol use disorder and a history of adverse childhood experiences.

## AUTHORS' CONTRIBUTIONS

Designed the current research: CT, HT, SG, SVK. Analysed the data: CT and HT. Supported the data analysis: YG, MS, TD. Wrote the first draft of the manuscript: CT. Interpreted the data: CT and HT. Conceived and designed the original study: SG, SVK and FK. Procured the funding of the original study: FK and SVK. Commented on the manuscript and provided intellectual input: all authors.

## CONFLICT OF INTEREST STATEMENT

None to declare.

## CLINICALTRIALS.GOV REGISTRATION IDENTIFIER

NCT03758053.

## STATEMENT OF ETHICS

This study was evaluated and approved by the ethics committee of the Medical Faculty Mannheim of the University of Heidelberg (ethics approval number: 2018‐560N‐MA), and conducted in accordance with the Declaration of Helsinki.

## Supporting information


**Table S1.** Exclusion criteria.
**Table S2.** Descriptive statistics of KERF subscales.
**Figure S1.** Average severity of KERF40 sum, abuse and neglect scores for ages 3 to 17 in the AUD group.
**Figure S2.** Categorical severity for each CTQ subtype in the AUD group.
**Figure S3.** Categorical severity for each CTQ subtype in the HC group.
**Figure S4.** Flow diagram of the study.

## Data Availability

The data that support the findings of this study are available from the corresponding author upon reasonable request.
