# Supplementary material for: The association between adverse childhood experiences and alterations in brain volume and cortical thickness in adults with alcohol use disorder
Source: Addict Biol. 2024 Sep 19;29(9):e13438. doi: 10.1111/adb.13438 (PMC11413060; doi:10.1111/adb.13438)
Supplement: Supplementary file 1 — Table S1. Exclusion criteria. Table S2. Descriptive statistics of KERF subscales. Figure S1. Average severity of KERF40 sum, abuse and neglect scores for ages 3 to 17 in the AUD group. Figure S2. Categorical severity for each CTQ subtype in the AUD group. Figure S3. Categorical severity for each CTQ subtype in the HC group. Figure S4. Flow diagram of the study. [file ADB-29-e13438-s001.docx]

**Supplementary material: “The association between adverse childhood experiences and alterations in brain volume and cortical thickness in adults with alcohol use disorder”**

**Table S1.** Exclusion criteria

- Withdrawal of the declaration of consent
- Exclusion criteria for an MRI scan (e.g., pregnancy, metal implants)
- Severe internal, neurological and psychiatric comorbidities (e.g., schizophrenia)
- Pharmacotherapy with psychoactive substances within the last 14 days (except treatment with SSRI/SNRIs for at least 28 days)
- Axis-I disorder according to ICD-10 and DSM 5 (except tobacco and alcohol use disorder, substance abuse with less than 2(11) criteria according to DSM-5, mild depressive episode, adaptation disorder and specific phobia within the last 12 months)
- positive urine drug screening (cannabis, amphetamine, opiates, benzodiazepines, cocaine)
- withdrawal symptoms (CIWA-AR^1^ > 7)
- intoxication at time of investigation (breathalyzer > 0.3‰)
- suicidal tendency or potential danger for others
- Healthy participants reporting a risky amount of alcohol consumption (alcohol/day ≥ 12 g (female), 24 g (male) on up to 5 days/week) or more than minimal severity of ACE (cut-off score of 8 [2*1+3*2])

1. Sullivan JT, Sykora K, Schneiderman J, et al. Assessment of alcohol withdrawal: the revised clinical institute withdrawal assessment for alcohol scale (CIWA-Ar). *Br J Addict* 1989;84(11):1353-7. doi: 10.1111/j.1360-0443.1989.tb00737.x

**Table S2.** Descriptive statistics of KERF subscales

|  | HC  Mean  (SD) | AUD  Mean  (SD) | Statistics |
| --- | --- | --- | --- |
| N | 28 | 35 |  |
| KERF PEA | 0.7 (1.6) | 2.8 (2.8) | **T(54.7) = -3.71, p < .001** |
| KERF PEAS | 0.0 (0.0) | 0.6 (1.8) | T(34) = -1.89, p = .067 |
| KERF PEER | 1.2 (2.3) | 4.1 (3.7) | **T(58.3) = -3.82, p < .001** |
| KERF WITS | 0.6 (1.7) | 2.2 (3.2) | **T(54.6) = -2.44, p = .018** |
| KERF PPA | 0.9 (2.1) | 3.4 (3.0) | **T(59.9) = -3.98, p < .001** |
| KERF EN | 0.7 (1.5) | 3.7 (3.7) | **T(47.2) = -4.41, p < .001** |
| KERF PN | 0.3 (1.0) | 0.7 (1.7) | T(57.9) = -1.30, p = .199 |
| KERF WITP | 0.4 (1.1) | 1.2 (2.6) | T(48.4) = -1.76, p = .085 |
| KERF SEXA_H | 0.0 (0.0) | 0.2 (1.0) | T(34) = -1.41, p = .169 |
| KERF SEXA_O | 0.3 (0.6) | 0.4 (1.1) | T(61) = -.507, p = .614 |

Note. *Significant group differences are highlighted in bold. SD = Standard Deviation; g = grams; N = sample size; PEA = Parental Emotional Abuse; PEAS = Physical and Emotional Abuse by Siblings; PEER = Physical and Emotional Abuse by Peers; WITS = Witnessed Violence towards Siblings; PPA = Parental Physical Abuse; EN = Emotional Neglect; PN = Physical Neglect; WITP = Witnessed Violence towards Parents; SEXA_H = Sexual Abuse by a Member of the Household; SEXA_O = Sexual Abuse by Others Not Living in the Same Household*

**Figure S1.** Average severity of KERF40 sum, abuse and neglect scores for ages 3 to 17 in the AUD group.


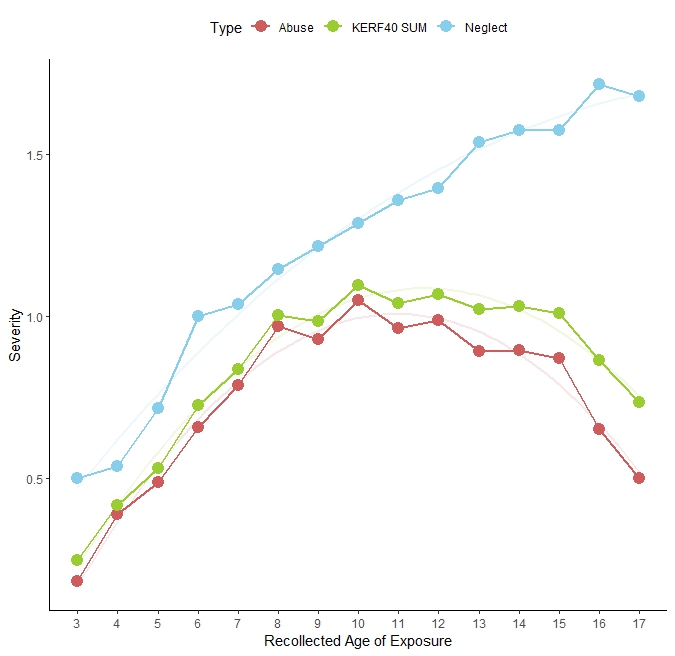


**Figure S2.** Categorical severity for each CTQ subtype in the AUD group.


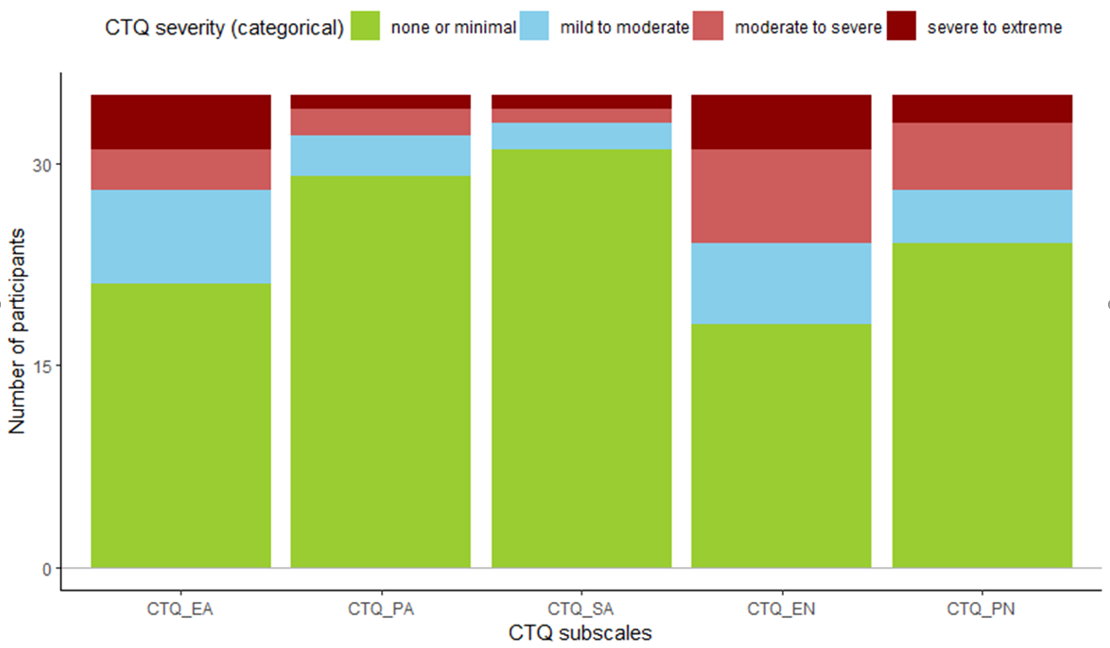


Note. *EA = Emotional Abuse; PA = Physical Abuse; SA = Sexual Abuse; EN = Emotional Neglect; PN = Physical Neglect*

**Figure S3.** Categorical severity for each CTQ subtype in the HC group.


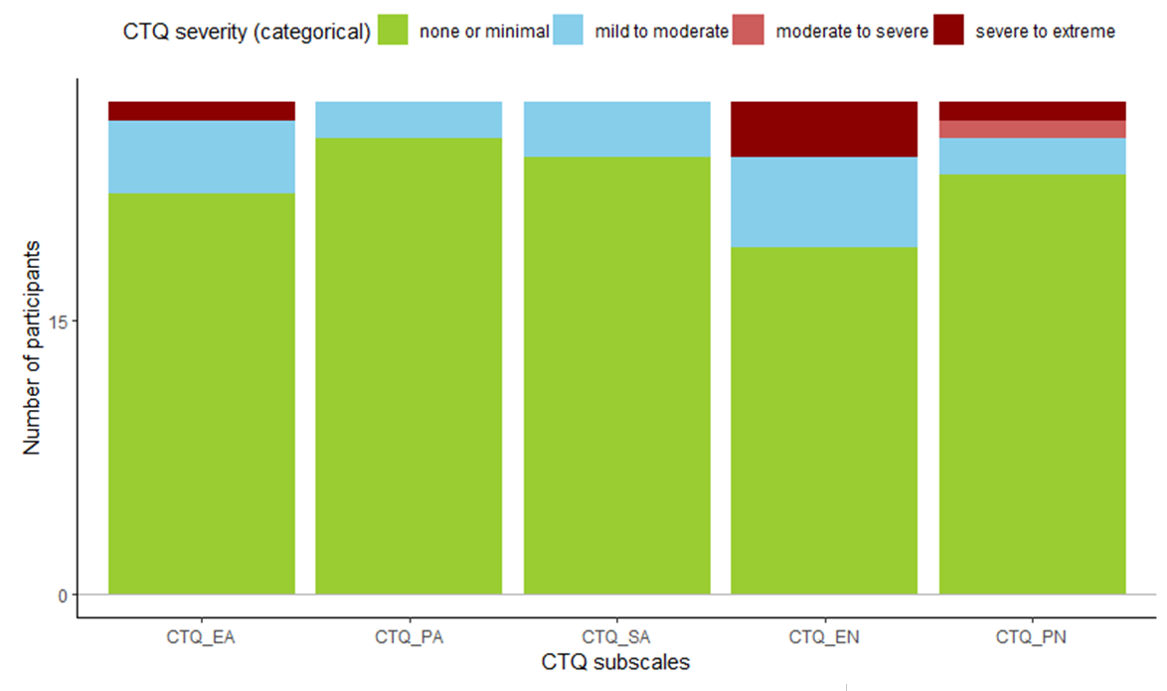


Note. *EA = Emotional Abuse; PA = Physical Abuse; SA = Sexual Abuse; EN = Emotional Neglect; PN = Physical Neglect*

**Figure S4.** Flow diagram of study.


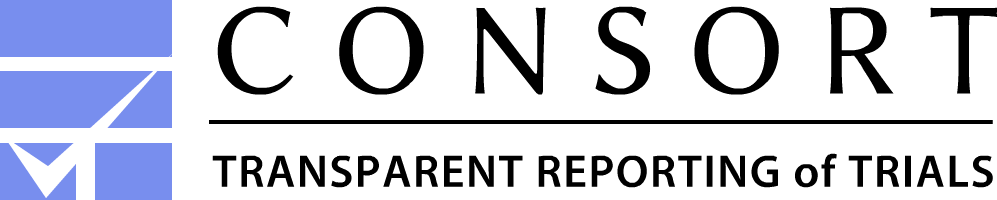


## Enrollment

Response (n= 221)

No interest, exclusion criteria (n= 82)

Analyzed (n = 35)

Excluded (n = 2) *due to*

- Brain abnormality (n = 1)
- MRI termination due to claustrophobia (n = 1)

Analyzed (n = 28)
Excluded (n = 2) *due to*

- Quality concerns (n = 1)
- MRI termination due to technical problems (n = 1)

No response, no interest, exclusion criteria (n= 71)

Screening and telephone interview (n= 152)

## Analyses

## MRI

## Allocation

Exclusion (n= 1)

Dropout before MRI assessment (n= 1)

Allocated to control group (n= 31)

Allocated to study group (n= 37)

Baseline (n= 69)

Dropout before MRI assessment (n= 0)
